# Numerically stable form factor of any polygon and polyhedron

**DOI:** 10.1107/S1600576721001710

**Published:** 2021-03-25

**Authors:** Joachim Wuttke

**Affiliations:** a Forschungszentrum Jülich GmbH, Jülich Centre for Neutron Science (JCNS) at Heinz Maier-Leibnitz Zentrum (MLZ), Lichtenbergstrasse 1, 85748 Garching, Germany

**Keywords:** form factors, polyhedra, Fourier shape transform

## Abstract

Coordinate-free expressions for the form factors of arbitrary polygons and polyhedra are derived using the divergence theorem and Stokes’s theorem. Apparent singularities, all removable, are discussed in detail. Cancellation near the singularities causes a loss of precision that can be avoided by using series expansions. An important application domain is small-angle scattering by nanocrystals.

## Abstract 2

Coordinate-free expressions for the form factors of arbitrary polygons and polyhedra are derived using the divergence theorem and Stokes’s theorem. Series expansions are used to ensure numeric precision close to apparent singularities.

## 1. Introduction   

### 1.1. Overview   

The term ‘form factor’ has different meanings in science and in engineering. Here, we are concerned with the form factor of a geometric figure as defined in the physical sciences, namely the Fourier transform of the figure’s indicator function, also called the ‘shape transform’ of the figure.

This form factor has important applications in the emission, detection and scattering of radiation. Two-dimensional shape transforms are used in the theory of reflector antennas (Lee & Mittra, 1983 ▸). The three-dimensional form factors of the sphere and the cylinder go back to Lord Rayleigh (1881 ▸). Shapes of three-dimensional nanoparticles are investigated by neutron and X-ray small-angle scattering (Hammouda, 2010 ▸). Particles grown on a substrate (Henry, 2005 ▸) develop many different shapes, especially polyhedral ones, as observed by grazing-incidence neutron and X-ray small-angle scattering (GISAS, GISANS, GISAXS) (Renaud *et al.*, 2009 ▸). Large collections of particle shape transforms have therefore been derived for and implemented in GISAS software (Lazzari, 2006 ▸; Pospelov *et al.*, 2020 ▸); another GISAS software package uses surface triangulation for computing approximative form factors (Chourou *et al.*, 2013 ▸). For attempts at direct reconstruction of polyhedral shapes from scattering patterns see the article by Engel & Laasch (2020 ▸) and literature cited therein.

In this paper, we derive a numerically stable algorithm for computing the form factor of any polygon or polyhedron, as implemented in the GISAS software *BornAgain* (Pospelov *et al.*, 2020 ▸). Originally, this algorithm was documented in a terse mathematical note (Wuttke, 2017 ▸). In the present paper, derivations and results have been simplified, the material has been completely reorganized for better readability, and additional literature is taken into account.

### 1.2. Different ways to compute form factors   

The form factor of a three-dimensional solid body 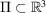 is 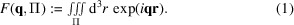
In most applications, the wavevectors **q** are real. In GISAS, however, the incident and scattered radiation may undergo substantial absorption, which can be modeled by an imaginary part of **q**. Therefore, we admit complex wavevectors 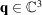.

For any polyhedron, (1) can be evaluated analytically by successive integration in the three coordinates. This is straightforward for a cuboid with edges along the coordinate axes. In most other cases, the algebra is cumbersome, and the resulting expressions are complicated and unattractive in that they do not reflect symmetries of the underlying shape. Striking examples are the form factors of the Platonic solids worked out in a *tour de force* by Li *et al.* (2011 ▸).

It is therefore preferable to derive a coordinate-free solution of (1) that expresses the form factor of a generic polyhedron in terms of its topology and vertex coordinates. This has been undertaken in different ways by Senesi & Lee (2015 ▸), Croset (2017 ▸) and Wuttke (2017 ▸). Senesi & Lee (2015 ▸) decomposed the polyhedron into pyramids and wrote the polyhedral form factor as the sum of the pyramidal form factor evaluated at different rotated **q**. Croset (2017 ▸) decomposed the polyhedron into simplexes, as explored previously by Lien & Kajiya (1984 ▸) and most recently by Li & Xie (2020 ▸), for the integration of multinomials. Following Wuttke (2017 ▸), we here present a different derivation that is based on use of the divergence theorem and Stokes’s theorem to reduce the volume integral to integrals over polygonal faces, and further reduce these surface integrals to line integrals over straight edges. This approach has been previously used for the integration of polynomials (Cattani & Paoluzzi, 1990 ▸; Bernadini, 1991 ▸) and for the computation of inertia moments (Mirtich, 1996 ▸).

Applications to nanoparticle assemblies typically require some averaging over particle sizes or/and orientations. How to compute these averages efficiently and with sufficient accuracy is an interesting and important question, which however is beyond the scope of the present work.

### 1.3. Singularities and asymptotes   

All analytical expressions for polyhedral form factors, derived by whatever method, contain denominators that vanish at **q** = 0. Croset (2017 ▸) suggested, and we will confirm, that the degree of this singularity is closely related to the asymptotic envelope of *F*(**q**, Π) for large *q*, which goes as *q* ^−1^, *q* ^−2^ or *q* ^−3^ depending on whether **q** is perpendicular to a face or an edge or points in an off-symmetric direction.

However, there is nothing fundamental about the singularities at **q** = 0: From the definition (1) in conjunction with Leibniz’s integral rule of differentiation we see that *F*(**q**, Π) is infinitely many times differentiable for all 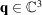; therefore *F* is a holomorphic function of each of the Cartesian components of **q**; therefore any apparent singularity is removable. Croset (2018 ▸) rederived the asymptotic envelopes by classifying the endpoint singularities of the section normal to **q** as function of height. In Section 3.6, we obtain them directly from our form factors.

The main purpose of this paper is to overcome numeric instabilities for small *q* and *q*_∥_. The latter is the wavevector component in the plane of a polygonal face. We will explain how rounding errors can grossly distort form factors when *q* or *q*_∥_ is of the order of ε/*a*, where ε is the machine precision and *a* is a typical edge length.

At this point the reader may wonder whether wavevectors with extremely small, but nonzero, *q* or *q*_∥_ have any practical importance. If wavevectors were drawn at random from an entire Brillouin zone, then the chance of ever hitting numerically problematic values would indeed be negligible. Often, however, **q** is chosen along a face normal. Roundoff errors then easily yield a tiny nonzero *q*_∥_, which causes huge, and symmetry breaking, errors in the form factor. Actually, this entire study started from the unexpected discovery of such artifacts in conventionally computed form factors.

## 2. Polygon form factor   

### 2.1. Notation   

A flat polygon Γ, embedded in three-dimensional space, shall be specified by its *J* vertices **V**_*j*_ (*j* = 1, …, *J*). Vertex indices shall be understood modulo *J* so that **V**_0_ ≡ **V**_*J*_. With this convention, the vertex sequence forms the closed loop ∂Γ. Edge *j* of the polygon is a straight line from **V**_*j*−1_ to **V**_*j*_. In most of this work it is more advantageously specified through its position 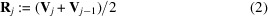
and mid-to-end vector 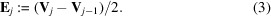
The normal vector 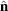 of the plane spanned by **V**_*j*_ shall be oriented such that ∂Γ has the winding number +1 (fulfills the right-hand rule with respect to 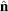). The oriented plane characterized by 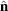 induces a decomposition of any vector 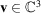 into a component perpendicular to the plane, 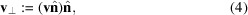
and an in-plane component, 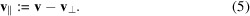
This decomposition will be applied to position vectors **r** and to wavevectors **q**. The oriented plane is fully specified by its normal vector 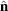 and its distance from the origin, *r*_⊥_.

Complex conjugation is denoted by a superscript asterisk. The absolute value of a complex vector is written 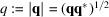.

Note that the in-plane unit vector 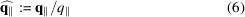
differs from the in-plane component 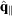 of the unit vector 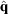. In this work, we shall only use 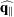 and 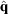, not 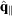.

The triple product is denoted 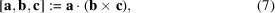
with the standard operators dot (·) for the scalar product and cross (×) for the vector product. Between adjacent vector symbols, as in the parentheses in (4), we omit the dot.

The cardinal sine function 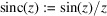 has the analytic continuation 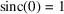. The numeric implementation for |*z*| → 0 is unproblematic: as 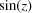 has full floating-point accuracy, so has 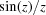.

### 2.2. Form factor   

We define the form factor of a flat figure Γ, embedded in three-dimensional space, as 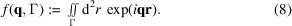

Proposition The form factor (8) of a flat *J*-gon Γ is 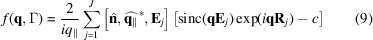
for *q*_∥_ ≠ 0, with notations from Section 2.1, and with an arbitrary constant *c* that can be chosen for computational convenience. The value at *q* = 0 is the area of Γ, 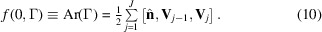
Values at *q* ≠ 0, *q*_∥_ = 0 can be obtained from 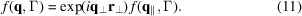

Proof For any vector field **G**, we have Stokes’s theorem: 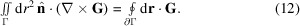
To prove (9), we choose 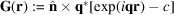. The left-hand side of (12) is 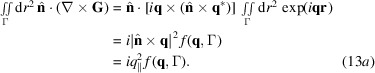
The right-hand side of (12) is 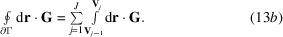
Each edge can be written as a parametric curve 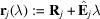 so that
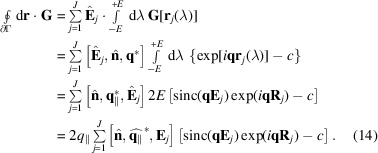
With *q*_∥_ ≠ 0, we obtain (9). Equation (11) follows directly from (8) and the fact that Γ is a flat figure with constant *r*_⊥_. To prove (10), we use Stokes’s theorem (12) with 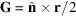. □

### 2.3. Remarks and example   

A closed expression for the form factor of the polygon has long since been known (Lee & Mittra, 1983 ▸, equation 6). A more symmetric expression was obtained by Croset (2017 ▸) (equation 4, where 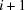 and 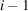 should be swapped). In our notation, it reads 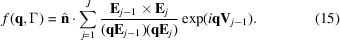
The equivalence with our equation (9) is proven in Appendix *A* [App appa]. Equation (15) is esthetically more pleasing than (9), but (15) is problematic for computer implementation and ill suited for the theoretical study of singularities, because for each *j* there are two **q** planes for which the denominator vanishes.

Equation (10) is well known as the ‘surveyor’s formula’. The standard proof uses triangular tessellation (Braden, 1986 ▸). See Appendix *B* [App appb] for a derivation of (10) from the *q*_∥_ → 0 limit of (9).

**Figure 1:**
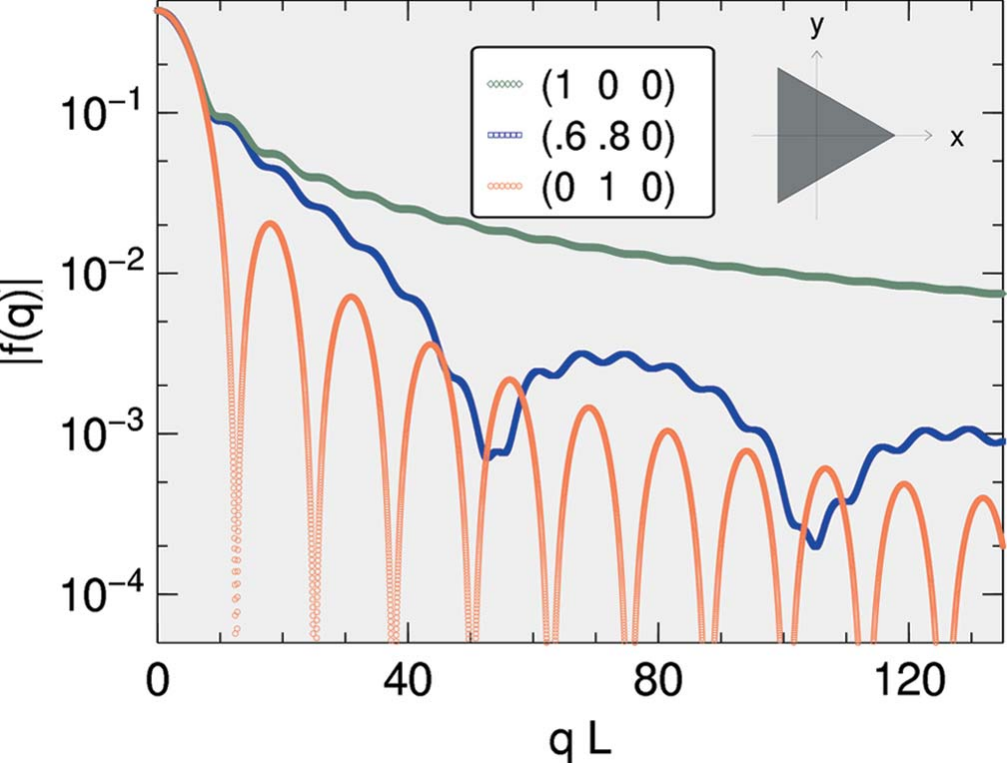
Modulus of the form factor of an equilateral triangle as function of wavenumber *q* for three different wavevector directions 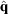. The triangle lies in the *xy* plane and has a symmetry axis along **x**. The center of gravity is at the origin. The edge length is *L* = 1.

Fig. 1 ▸ shows for an equilateral triangle how strongly the form factor, plotted as |*f*(*q*)| versus *q*, varies with the wavevector direction 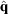.

### 2.4. Removable singularity   

**Figure 2:**
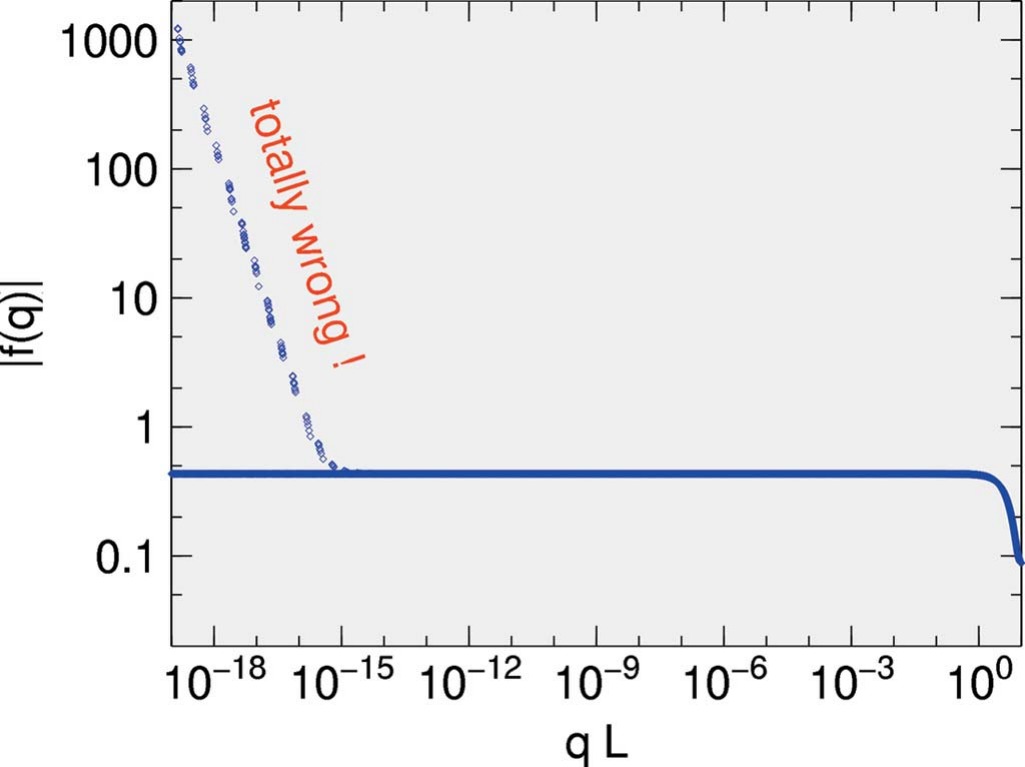
Same form factor modulus as in Fig. 1 ▸, for 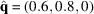, now in a double logarithmic plot, computed in double-precision arithmetic according to the closed expression (9) with *c* = 0. For some small *q*, the results are totally wrong owing to imperfect cancellation in the leading-order term that ought to vanish.

The closed expression (9) for the polygonal form factor *f*(**q**, Γ) has a singular factor 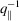. As discussed in Section 1.3, the definition of *f* guarantees its analyticity. So we know that the apparent singularity at *q*_∥_ → 0 is removable. We also know the value at *q*_∥_ = 0, given by (10) and (11). Nonetheless, the presence of a divergent factor may cause numeric instabilities for small values of *q*_∥_. To investigate this more closely, let us write (9) as 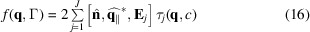
with the function 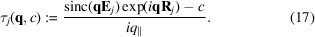
The constant *c* can be chosen differently for different **q**. At large *q*_∥_, 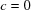 prevents roundoff errors in the numerator of (17). For small *q*_∥_, 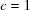 is the better choice. To see this, we expand τ_*j*_ as 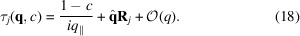
The leading, *j*-independent term in the expansion, with the apparent singularity (*iq*_∥_)^−1^, contributes nothing to the sum in (16), whatever the value of *c*, because 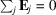. This, however, holds only in exact arithmetics; in a floating-point implementation, roundoff errors can make the sum nonzero. For *q*_∥_ → 0, any such error outgrows all other terms in the expansion (Fig. 2 ▸). Therefore, in the small-*q*_∥_ regime, the only sensible choice is *c* = 1, which lets the leading term vanish.

Unfortunately though, the subtraction of 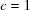 can cause roundoff errors in the numerator of (17). As a straightforward remedy, we compute τ_*j*_ for small *q*_∥_ from its series expansion 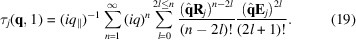
As a consistency check, we note that the limit 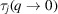, at constant in-plane direction 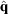, is the 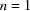 term 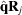. Plugging this value into (16), we recover the surveyor’s formula (10). The algebra is quite lengthy and therefore relegated to Appendix *B*.

**Figure 3:**
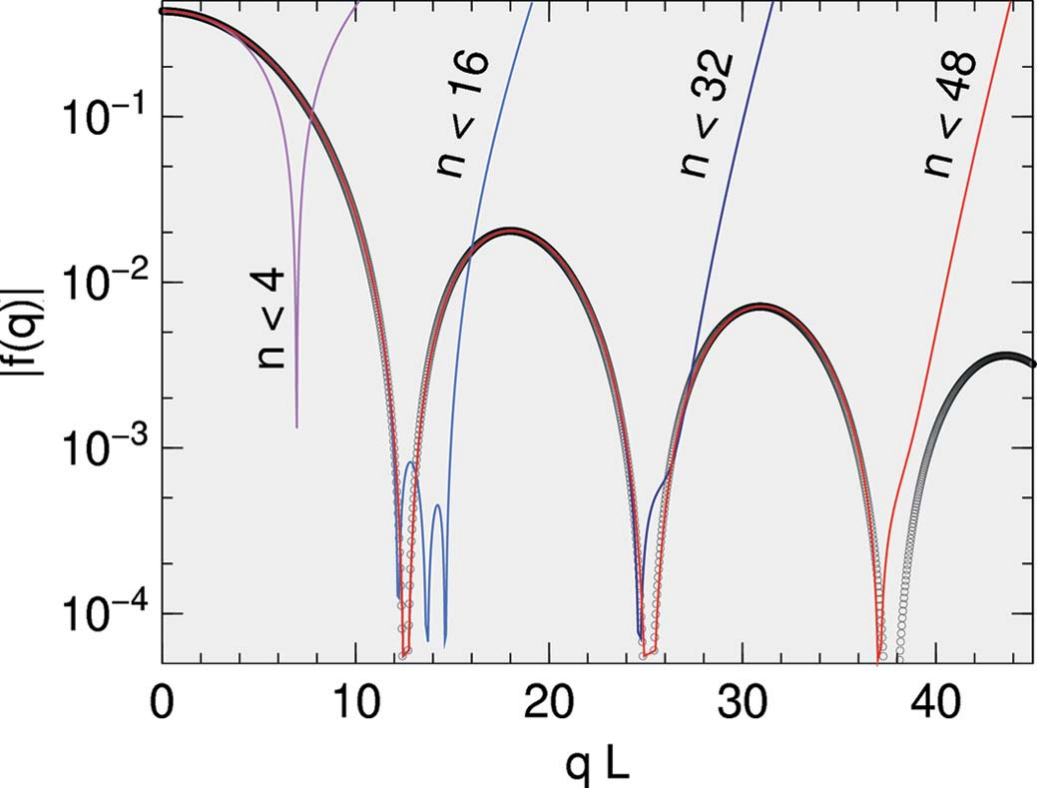
Modulus of the form factor of the equilateral triangle of Fig. 1 ▸, as function of wavenumber *q* for wavevector direction 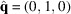. The black chain is computed using the analytical expression (9). The colored curves are computed using the series expansion (19) up to the indicated order *n*.

Fig. 3 ▸ compares the series expansion with the closed expression (9). The series expansion works well even beyond the first minima in *f*(|*q*|). In practice, the series expansion is only needed for 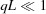, and therefore only a few expansion orders are needed to keep errors close to machine precision.

### 2.5. Polygon with inversion center   

Computations can be simplified, and the numeric instability at *q*_∥_ → 0 avoided, if a polygon has a perpendicular twofold symmetry axis (Schoenflies group *S*_2_) or, equivalently, an inversion center at **ρ**. The form factor has the symmetry *f*(**q**_⊥_ + **q**_∥_, Γ) = *f*(**q**_⊥_ − **q**_∥_, 2**ρ** − Γ). As the number of vertices is even, we can write *J* = 2*J*′ and use 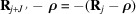 and 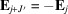. For *q*_∥_ ≠ 0, the form factor is 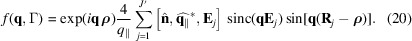
In contrast to (9), the summand in (20) has no constant contribution but is linear in *q*. There is no cancellation for *q*_∥_ → 0 and no need to use a series expansion for the accurate computation of *f*.

## 3. Polyhedron form factors   

### 3.1. Notation and parameterization   

An orientable polyhedron Π shall be specified by its *K* polygonal faces Γ_*k*_ (*k* = 1, …, *K*). For each face Γ_*k*_, the normal 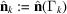 shall point to the outside of Π; this then determines the order of the vertices in the sequence 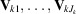.

**Figure 4:**
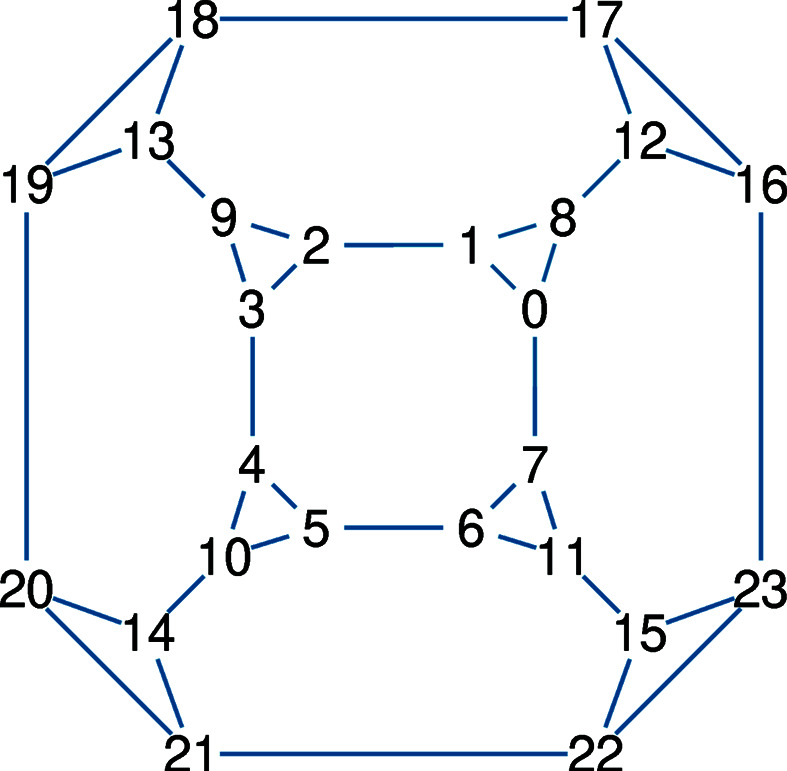
Schlegel diagram of a facetted cube. This representation of polyhedral topology can help to assign vertex indices in a systematic way, which then facilitates the coding of 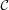 and 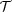. The topology array 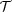 has elements (0, 1, 2, 3, 4, 5, 6, 7), (0, 8, 1) *etc*. The coordinate array 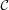, parameterized on lengths *a* and *b*, has elements (*a*, *b*, *a*), (*b*, *a*, *a*), (−*b*, *a*, *a*) *etc*.

In a computer implementation, the topology and geometry of a polyhedron can be specified through two arrays: Array 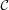 holds one coordinate triple **V**_α_ for each of the polyhedron’s vertices. Array 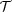 holds one array γ_*k*_ for each of the polyhedron’s faces Γ_*k*_; γ_*k*_ holds the global indices α_*kj*_ of the vertices that belong to face Γ_*k*_, such that 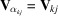. In short, array 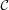 holds the coordinates and array 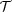 holds the topology of the polyhedron. For the latter, Schlegel diagrams (Fig. 4 ▸) provide a helpful visualization. In physical simulations, 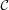 is typically generated by a parametric function, whereas 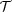 is static. An assertion in the computer code should ensure that all faces are planar for any geometry parameters.

Additionally, it is advantageous to foresee boolean parameters to indicate the presence or absence of inversion centers. One needs one such parameter for the entire polyhedron and one for each of its polygonal faces.

### 3.2. Form factor  

Proposition If *q* ≠ 0, then the form factor (1) of a *K*-hedron Π is 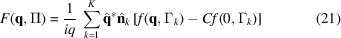
with an arbitrary constant *C* that can be chosen for computational convenience. Otherwise, for *q* = 0, the form factor is just the volume of Π, 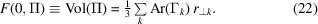

Proof For a polyhedron, the divergence theorem takes the form 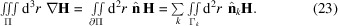
With the choice 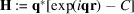, this yields 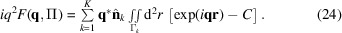
With the notation (8), this proves (21). With the choice 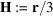, we obtain the volume formula (22). □

Similar to *c* in Section 2, the constant *C* can and should be chosen differently for different **q** domains. At large *q*, the best choice is *C* = 0. The small-*q* case is discussed in Section 3.3.

The volume formula (22) has previously been derived by tetrahedral tessellation (Comessatti, 1930 ▸, Cap. II, §3, III 171).

To see the equivalence of (12) with equation 15 of Croset (2017 ▸), we let *C* = 0, take *f*(**q**, Γ) from (15) and use the fact that **E**_*j*−1_ × **E**_*j*_ is colinear with 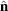.

### 3.3. Removable singularity   

The closed expression (21) for the polyhedral form factor *F*(**q**, Π) contains two removable singularities: the explicit factor *q* ^−1^, and the factor 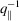 contained in the polygonal form factors *f*(**q**, Γ_*k*_). For the case that only *q*_∥_, but not *q*, is close to 0, we rely on the numerically stable computation of *f* derived in Section 2.4. Here we address the case *q* → 0.

**Figure 5:**
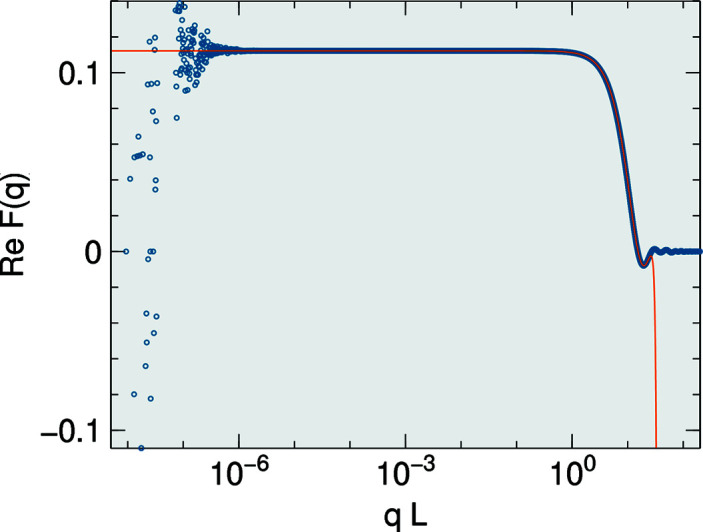
Modulus of the form factor of a truncated tetrahedron (trigonal pyramidal frustum). The base is an equilateral triangle in the *xy* plane, oriented so that an edge points in the **y** direction, with edge length *L* = 1; the dihedral angle is 72°; the height *H* = *L*/2. The plot shows 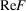 versus *q* for the off-symmetric direction 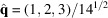. Blue spots are computed using the analytical expressions (9) and (21). For *qL* < 10^−6^, roundoff errors dominate. The orange line is computed according to (26) with summation (28) up to *n* = 19.

In analogy to Section 2.4, it is sufficient to invoke analyticity to convince ourselves that the singularity of *F* is removable. The value of *F* in the limit *q* → 0 is just the volume of Π. The expansion of (21) starts with 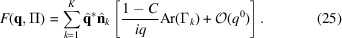
The leading, apparently singular term is identically zero because 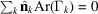. This, however, holds only in exact arithmetics; in a floating-point implementation, roundoff errors can make the sum nonzero. For *q* → 0, any such error outgrows all other terms (Fig. 5 ▸). Therefore, in the small-*q* regime, the only sensible choice is *C* = 1, which lets the leading term vanish.

Unfortunately though, this can lead to roundoff errors in the difference *f*(**q**) − *f*(0) in (21). As a remedy, we compute the form factor from a series expansion as follows: Combine (21) and (16) to write the form factor as 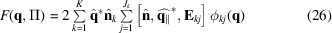
with the function 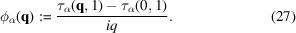
The constant τ_α_(0, 1) neutralizes the first term in the expansion (19) of τ_α_(**q**, 1) so that 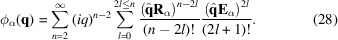
Fig. 5 ▸ shows that there is good overlap between the domains of the closed expression and the series expansion.

### 3.4. Polyhedron with inversion center   

If a polyhedron has an inversion center at **ρ** (Schoenflies group *C*_*i*_), then the form factor has the symmetry *F*(**q**, Π) = *F*(−**q**, 2**ρ** − Π). As the number of faces is even, we can write *K* = 2*K*′. We require that faces numbered *k* and *k* + *K*′ be opposite to each other. We use 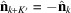 to write the form factor as 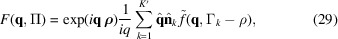
where 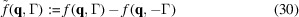
is the form factor of a pair of opposite faces. The symmetry *f*(**q**, −Γ) = *f*(−**q**, Γ) allows some economy in computing *F* from the generic closed expression.

In the small-*q* case, the expansion (26) is symmetrized as 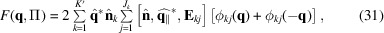
and in consequence in (28) the terms with odd *n* cancel.

### 3.5. Prism   

For a prism Π = {**r** | **r**_∥_ ∈ Γ_∥_, |*r*_⊥_| < *h*/2} a much simpler solution is available. We return to the definition (1). Applying Fubini’s theorem to factorize the triple integral from the onset into an integral (8) over the base Γ_∥_ of the prism and an integral along the normal direction 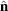, we obtain the form factor 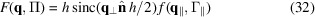
for all **q**. Thanks to the sinc function in (32), there is no singularity in *q*_⊥_ and therefore no series expansion is needed for *q*_⊥_ → 0.

### 3.6. Asymptotic envelopes   

We now come back to the asymptotic power-law envelopes for large *q* discussed in Section 1.3. A cube Π with side lengths *L*, centered at the origin and oriented along the coordinate axes, has the form factor 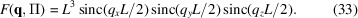
For large *q* it has the asymptotic envelope |*F*| ≤ 8/|*q*_*x*_ *q*_*y*_ *q*_*z*_|, which goes as *q* ^−3^ for fixed direction 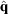, provided none of the three components 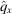, 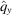, 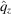 is zero. If 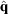 is perpendicular to one of the edges of the cube, then one of the three sinc functions has the fixed argument 0 and value 1. And if 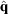 is perpendicular to one of the faces of the cube, then (33) has two constant factors 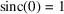. As Croset (2017 ▸) has worked out, these observations can be generalized to any polygon. Within our present formalism, this can be confirmed as follows.

For *q* ≠ 0, the form factor (21) of any *K*-hedron Π is limited by 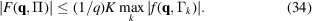
For *q*_∥_ ≠ 0, the form factor (9) of any *J*-gon Γ is limited by 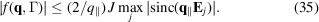
For **q** **E**_*j*_ ≠ 0, the sinc function in (35) is limited by 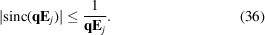
So if all the above conditions are fulfilled, then the polyhedron form factor *F* has an asymptotic envelope ∼*q* ^−3^. If there is any edge perpendicular to **q**, then (36) is not applicable, and 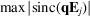 takes the *q*-independent value 1, so that the envelope of *F* goes with *q* ^−2^. If there is any face perpendicular to **q**, then (35) is not applicable, and 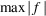 in (34) takes the *q*-independent value 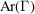; so the envelope of *F* goes with *q* ^−1^.

## 4. Concluding remarks   

### 4.1. Implementation   

Code for computing the form factor of any polygon or polyhedron, based on all the above, has been implemented as part of the open-source GISAS simulation package *Born­Again* (Pospelov *et al.*, 2020 ▸). All floating-point numbers, internal and external, have double precision. A summary of the algorithm is given in Appendix *C* [App appc].

The code underwent extensive tests for internal consistency and for compatibility with conventional form factor formulae. Checks of *BornAgain* against the reference code *IsGISAXS* (Renaud *et al.*, 2009 ▸; Lazzari, 2006 ▸) have been documented by Pospelov *et al.* (2020 ▸). In the following, we describe form factor consistency checks that have been permanently added to the *BornAgain* unit tests.

### 4.2. Tests   

The internal consistency tests address symmetry, specialization and continuity. Symmetry tests are performed for particle shapes that are invariant under some rotation or reflection *R*. For a suite of wavevectors **q**, it is checked that the relative deviation of form factors *F*(**q**) and *F*(*R* **q**) stays below a given bound.

The specialization tests address pairs of figures Π_1_, Π_2_ with different topologies that coincide for certain geometry parameters. For instance, if Π_1_ is a box with side lengths *a*, *b*, *c*, and Π_2_ a truncated cube with side length *a* and truncation length *t*, then the choices *a* = *b* = *c* and *t* = 0 reduce Π_1_ and Π_2_ to the same cube. For a suite of wavevectors **q**, it is checked that the relative deviation of form factors *F*(**q**, Π_1_) and *F*(**q**, Π_2_) stays below a given bound.

The continuity tests search for possible discontinuities due to a change in the computational method. They need special instrumentation of the code, activated through a CMake option and a precompiler macro. Under this option, additional variables tell us whether the analytical expression or the series expansion has been used in the latest form factor computation, and, if applicable, at which expansion order the summation was terminated. For a given direction 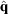, bisection is used to determine wavevectors where one of these variables changes. Then, the form factor *F* is computed for wavevectors slightly before and slightly after the transition, and it is checked that the relative step in *F* remains below a given bound.

All these tests are performed for a suite of particle shapes, for different wavevector directions 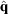 with different degrees of symmetry, for a logarithmically wide range of magnitudes *q* and for a range of complex phases.

### 4.3. Crossover metaparameters   

For large *q*, the polyhedral form factor is computed from (21) with *C* = 0. For small *q*, we use (26) with the expansion (28). Therefore, we need a heuristic metaparameter 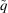 that determines which algorithm to use. For large *q*, it still can happen that *q*_∥_ is small. Therefore, a second metaparameter 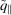 is needed to determine whether face form factors are computed from the closed expression (9) or from (16) with the expansion (19). As 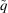 and 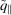 are dimensionless, the choice of algorithm is based on *qr* and *q*_∥_ *r*, where *r* is the radius of the circumscribing sphere of figure Π. Under a multitude of tests, we obtained the best results with 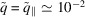.

### 4.4. Accuracy   

Currently, the bounds for maximum relative form factor discrepancies are 10^−11^ in symmetry tests, 6 × 10^−12^ in specialization tests and 6 × 10^−9^ in continuity tests. Discrepancies reaching the order of magnitude of these bounds are only observed for a few out of hundreds of thousands of test cases. Most often, errors are smaller than 10^−15^, *i.e.* a small multiple of the machine precision. Some of the larger discrepancies are compiler or processor dependent.

The cases of relatively large discrepancy that we have investigated so far all involve special wavevectors that make the integral (1) more symmetric than the underlying figure Π. Appendix *D* [App appd] presents one such case: a pyramid that acquires the inversion symmetry of a bipyramid if **q** lies in the base plane.

It remains to be seen whether such cases warrant closer attention and improved code. So far, we have not encountered a single **q**, Π combination where symmetry, specialization or continuity tests revealed numeric errors larger than single-precision machine error.

## 

## Appendix A Polygon form factor in the literature

As a complement to Section 2.3, we demonstrate the equivalence of our form factor (9) with the result (15) of Lee & Mittra (1983 ▸) and Croset (2017 ▸). We start from (9), choose *c* = 0 and expand the unit in-plane vector :

Expanding the sinc function, and using (2) and (3) to simplify , we obtain

Using , and hence **q** **E** = **q**_∥_ **E**,

Using , we obtain (15).

## Appendix B Polygon area as lowest expansion coefficient

As a complement to Section 2.4, we demonstrate the equivalence of two different expressions for the area of a polygon: the  limit of the generic form factor (9), and the surveyor’s formula (10). For any constant direction  with , we have from (16) and (19)

Splitting **q** = **q**_⊥_ + **q**_∥_, drawing the constant *q*_⊥_ *r*_⊥_ in front of the sum and using  we obtain

Inserting the definitions of **E**_*j*_ and **R**_*j*_,

Multiplying out and shuffling indices  for some terms under the sum gives

Using , we recover (10).

## Appendix C Algorithm summary

In this appendix, we summarize the algorithm for the computation of the form factor *F*(**q**; Π) as derived in this work. For clarity and brevity, we only consider a polyhedron Π without any inversion symmetry. For polyhedra with inversion symmetry, and for other details omitted here, see the actual implementation in the open-source code *BornAgain*.

Read the wavevector **q**, the topology , the vertex coordinates  and the symmetry flags (Section 3.1, ignored here). Discard faces with zero or negligible area. For each face, merge adjacent vertices with zero or negligible distance. Assert that all remaining faces are planar. Compute the circumscribing radius *r* of Π (Section 4.3). For each face Γ_*k*_, compute the circumscribing *r*_*k*_.

If *qr* < 10^−2^ (Section 4.3), then compute *F* according to (26) and (28), with changed summation order so that the outermost summation runs over the expansion power *n*. Terminate if the relative contribution of two subsequent expansion terms is less than ε = 2 × 10^−16^.

Otherwise (), compute *F* according to (21) with *C* = 0. This is a weighted sum of polygonal face form factors *f*(**q**, Γ_*k*_). They are computed as follows:

If *q*_∥_ *r*_*k*_ < 10^−2^, then compute *f* according to (16) and (19) with changed summation order so that the outermost summation runs over the expansion power *n*. Terminate if the relative contribution of two subsequent expansion terms is less than ε = 2 × 10^−16^.

Otherwise (*q*_∥_ *r*_*k*_ ≥ 10^−2^), compute *f* according to (9) with *c* = 0.

## Appendix D Additional symmetry at special wavevectors

As a complement to Section 4.4, we give one example of how special wavevectors cause extra symmetries in the integral (1) that lead to cancellation and roundoff errors. Consider a pyramid Π with a base Γ_0_ that has at least a twofold rotation axis, as considered in Section 2.5. Let  be the normal of Γ_0_, pointing towards the outside of Π. With (4) and (5) it induces the decomposition **r** = **r**_∥_ + **r**_⊥_.

Now consider a wavevector **q** in the plane of Γ_0_ so that *q*_⊥_ = 0. To see how this causes an extra symmetry in the integral (1), consider the bipyramid Π_2_ that is the union of Π and the mirror image of Π under reflection about the Γ_0_ plane,

Obviously, for the special in-plane **q** under consideration,

Thereby, the form factor integral (1) for *F*(**q**, Π) has the extra symmetries of the bipyramid Π_2_. The combination of the *S*_2_ symmetry assumed for Γ_0_ and the mirror symmetry that constitutes the bipyramid implies an inversion symmetry around the center of Γ_0_. As discussed in Section 3.4 this leads to cancellation of some terms. Unless these terms are removed from the implemented formulae, severe roundoff errors must be expected.
